# Extracellular miR-1246 promotes lung cancer cell proliferation and enhances radioresistance by directly targeting DR5

**DOI:** 10.18632/oncotarget.9017

**Published:** 2016-04-26

**Authors:** Dexiao Yuan, Jinping Xu, Juan Wang, Yan Pan, Jiamei Fu, Yang Bai, Jianghong Zhang, Chunlin Shao

**Affiliations:** ^1^ Institute of Radiation Medicine, Fudan University, Shanghai 200032, China

**Keywords:** extracellular mR-1246, exosomes, radioresistance, DR5 gene, bystander effect

## Abstract

MiRNAs in the circulation have been demonstrated to be a type of signaling molecule involved in intercellular communication but little is known about their role in regulating radiosensitivity. This study aims to investigate the effects of extracellular miRNAs induced by ionizing radiation (IR) on cell proliferation and radiosensitivity. The miRNAs in the conditioned medium (CM) from irradiated and non-irradiated A549 lung cancer cells were compared using a microarray assay and the profiles of 21 miRNAs up and down-regulated by radiation were confirmed by qRT-PCR. One of these miRNAs, miR-1246, was especially abundant outside the cells and had a much higher level compared with that inside of cells. The expressions of miR-1246 in both A549 and H446 cells increased along with irradiation dose and the time post-irradiation. By labeling exosomes and miR-1246 with different fluorescence dyes, it was found that the extracellular miR-1246 could shuttle from its donor cells to other recipient cells by a non-exosome associated pathway. Moreover, the treatments of cells with miR-1246 mimic or its antisense inhibitor showed that the extracellular miR-1246 could enhance the proliferation and radioresistance of lung cancer cells. A luciferase reporter-gene transfer experiment demonstrated that the death receptor 5 (DR5) was the direct target of miR-1246, and the kinetics of DR5 expression was opposite to that of miR-1246 in the irradiated cells. Our results show that the oncogene-like extracellular miR-1246 could act as a signaling messenger between irradiated and non-irradiated cells, more importantly, it contributes to cell radioresistance by directly suppressing the DR5 gene.

## INTRODUCTION

Radiotherapy treatment is widely used for the treatment of malignant tumors, however, radioresistance remains a major clinical obstacle to a successful cancer therapy and it may lead to poor prognosis of radiotherapy. It has been reported that lung cancer could effectively evade radiation-mediated cell killing by a number of strategies including radioresistance and tumor microenvironment mediated signaling [1, 2]. Among them, miRNAs responding to ionizing radiation (IR) may play an important role in the regulation of radiosensitivity [3]. Several studies have found that miRNAs are not only present inside of cells but also exist in a various of body fluids including plasma, serum, urine, saliva, milk and semen [4–6], and those miRNAs are commonly termed as extracellular miRNAs or circulating miRNAs. Extracellular miRNAs were found to be remarkably stable in plasma despite it containing a high RNase activity [4], suggesting that the circulating miRNAs are actively protected in the harsh extracellular environment. Associationwith exosomes is the dominant model of extracellular miRNA stably existing in different bio-fluids [7–10]. In addition, a significant portion of extracellular miRNAs stay in non-exosome associated structures including the complexes combined with argonaute 2 (AGO2) [11], nucleophosmin-1 (NPM1) [12] and high density lipoprotein (HDL) [13]. The findings of stable extracellular miRNAs in body fluid raises great interest in the physiological function of such external miRNAs.

Stable miRNAs in exosomes are thought to be an important mediator of intercellular signaling between different tumor cells and between tumor cells and its surrounding bystander stromal cells [10, 14–16]. The extracellular miRNAs that can function in recipient cells are named as shuttle miRNAs, it may convey specific information from its donor cells to other recipient cells and hence plays various physiological functions. Recent studies have suggested that the expressions of specific miRNAs in blood plasma could be used as biomarkers of radiation dosimetry [17, 18].

As a soluble signaling factor, miRNAs may also contribute to the radiation-induced bystander effect (RIBE). RIBE can be induced through the signaling communication between irradiated cells and non-irradiated cells [19, 20]. Numerous studies have confirmed that many kinds of diffusible signaling factors contribute to the generation of RIBE, including reactive oxygen species (ROS), reactive nitrogen species (RNS), cytochrome-c and cytokines [21–23]. Recently, it was reported that miR-663 and miR-21 were involved in the RIBE through a medium-mediated pathway [24, 25]. However, the characteristics of extracellular miRNA expressions responding to IR and the potential roles of miRNAs in regulating cell radiosensitivity are still largely unknown.

The present study revealed that the profiles of extracellular miRNAs could be significantly altered by IR, especially, miR-1246 in non-exosome-associated form was actively secreted into the culture medium and enriched outside of the lung cancer cells after IR. This extracellular miR-1246 could be taken up by other recipient cells and subsequently increased their survival and radioresistance by directly suppressing the death receptor 5 (DR5) gene expression.

## RESULTS

### Radiation alters the profiles of extracellular miRNAs

**Figure 1:**
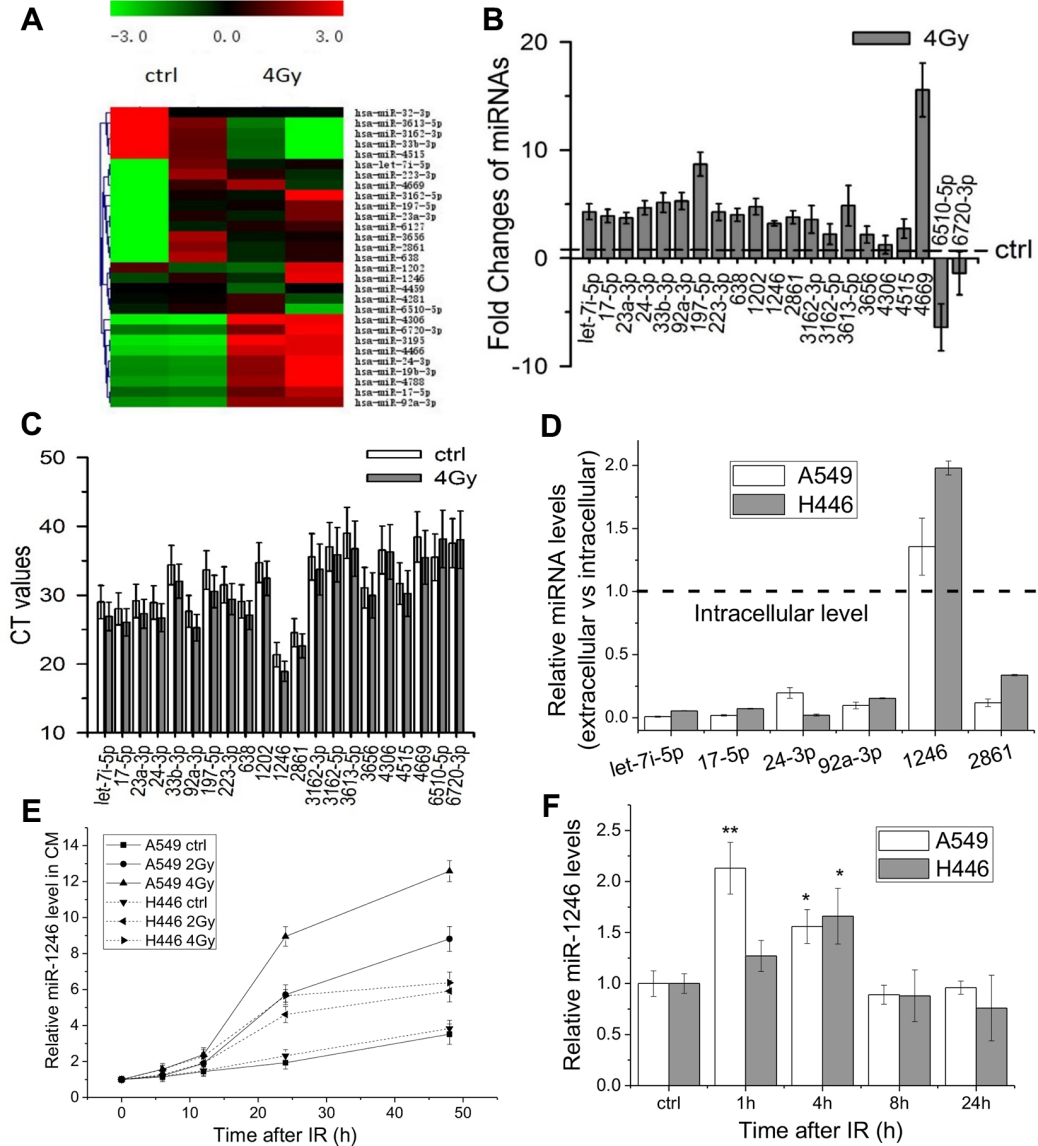
Radiation-induced alterations of extracellular miRNA expression profiles in lung cancer cells (**A**) Hierarchical clustering of differentially expressed miRNAs in the CM of A549 cells at 24 h after 0 Gy or 4 Gy X-ray irradiation. Red indicates higher expression, green indicates lower expression, and black means no expression difference. (**B**) Quantification of 21 differentially miRNAs in the above CM confirmed by qRT-PCR. Results are expressed as relative levels compared with non-irradiated controls and normalized to spike control (cel-miR-39). Each reaction was performed in triplicate. (**C**) The CT values of above 21 differentially miRNAs with qRT-PCR assay. (**D**) Ratio of extracellular miRNAs to intracellular miRNAs of A549 and H446 cells. Equal amounts of RNAs (20 ng) isolated from cells and CM were analyzed with qRT-PCR. (**E**) Relative levels of extracellular miR-1246 in the CM of A549 and H446 cells after indicated times (0, 6, 12, 24 or 48 h) of irradiation (0, 2, 4 Gy). (**F**) Relative expression levels of intracellular miR-1246 in A549 and H446 cells 1, 4, 8 or 24 h after 4 Gy irradiation. Data were presented as means ± SEMs of three independent experiments. *P < 0.05, **P < 0.01 compared to non-irradiated controls.

It has been known that radiation can alter gene expressions [26]. To determine which extracellular miRNA expressions could be significantly changed in lung cancer cells in response to IR, miRNAs in the conditioned medium (CM) collected from irradiated A549 cells at 24 h after 4 Gy irradiation were compared with that from non-irradiated control by using the Agilent Human miRNA microarray containing 2006 human miRNA probes. The heat map of its expression profiles was shown in Figure 1A. Although the expression levels of different miRNAs had some variability in duplicate experiments, a total of 17 up-regulated miRNAs (fold change ≥ 2) and 4 down-regulated miRNAs (fold change ≤ 0.5) were detected in the CM of irradiated cells compared with control. QRT-PCR was used to further confirm the expression profiles of these 21 IR-responsive extracellular miRNAs, and 19 of 21 miRNAs were found to be up-regulated (Figure 1B). Figure 1B also illustrates that let-7i-5p, miR-17-5p, −24-3p, -92a-3p, -1246, and -2861 were not only significantly up-regulated but also had high concentrations in the CM because they had low PCR cycle thresholds (CT < 30), especially, miR-1246 had the lowest CT value (Figure 1C). However, there were tiny inconsistent differences between the microarray and qRT-PCR analyses. The microarray assay suggested that the expression of miR-6720-3p was up-regulated by IR, but the qRT-PCR indicated that it was down-regulated. The reason for this inconsistence may due to the low abundance of miRNAs characterized by high cycle threshold (CT > 30), for instance, miR-3162, -3613-5p,-4306, -4669, 6720-3p and -6510-5p (Figure 1C).

### Kinetics of extracellular miR-1246 expression after IR

If a circulating miRNA plays a physiological function, its extracellular concentration should be higher than that inside of cells. Here we compared the levels of let-7i-5p, miR-17-5p, −24-3p, -92a-3p, -1246 and -2861 in the CM with those inside of cells. Figure 1D illustrates that, in both cell lines of A549 and H446, only miR-1246 outside of cells had a higher level than that inside of cells, and the expressions of other five extracellular miRNAs were quite lower, in general, 1%–20% of their intracellular contents. Therefore, miR-1246 might have some special functions and was selected for further investigation.

To know the dose- and time- responses of extracellular miR-1246 release, the expressions of miR-1246 in the active conditioned media (ACM) and control conditioned media (CCM) from irradiated A549 and H446 cells and their controls 6, 12, 24 and 48 h after 2 Gy and 4 Gy irradiation were detected with qRT–PCR. It was found that the level of extracellular miR-1246 in the CCM was increased along with the culture time from 0 to 48 h, which may due to the increase of cell number along with culture time since miRNAs can be actively released under normal physiological conditions [27, 28]. It was found that the level of extracellular miR-1246 in the ACM was much higher than that in the CCM, and it increased in a dose-dependent manner along with the culture time after irradiationCM (Figure 1E). In the present study, cel-miR-39 (25 fmol) was added to 1 ml CM as a spike control for the normalization of qPCR assay. Thus, based on the PCR Ct values of cel-miR-39 and miR-1246, the concentration of miR-1246 in the CM was calculated. For A549 cells, after 24 h of cell culture, the miR-1246 concentration in the CCM and the ACM of 2 Gy irradiated cells was 195 fmol/L and 527 fmol/L, respectively. After IR, the extracellular miR-1246 in the ACM was increased to 2.7-folds and 4.5-folds of control for 2 Gy and 4 Gy irradiated cells, respectively. The similar situation of miR-1246 generation occurred for H446 cells. Although the intracellular miR-1246 transiently increased after IR, it decreased along with cell culture time after irradiation (Figure 1F). Moreover, after irradiation, the changes of extracellular miR-1246 was remarkably greater than intracellular miR-1246. These results suggest that miR-1246 can be actively released from irradiated lung cancer cells to culture medium after IR.

### Extracellular miR-1246 exists in a non-exosome associated form

**Figure 2:**
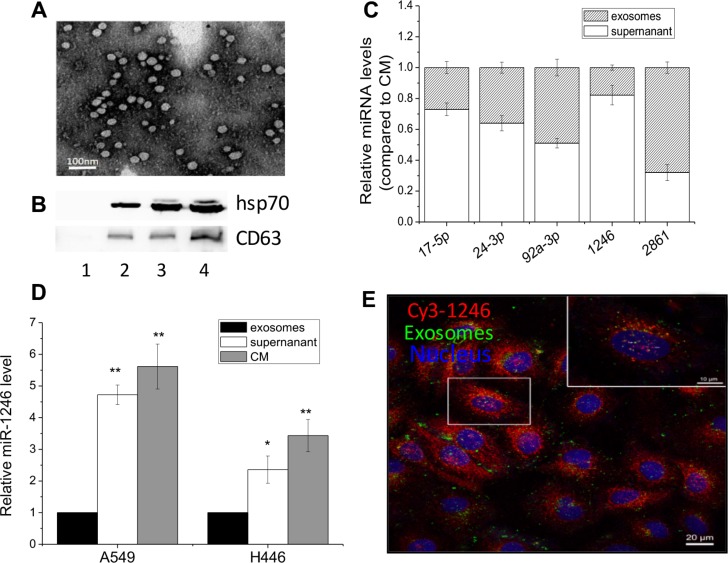
Extracellular miR-1246 exists in non-exosomes associated form (**A**) Exosomes released from A549 cells were observed under an electron microscopy. (**B**) Hsp70 and CD63 protein expressions in the exosomes purified from the medium with depleted exosomes FBS (lane 1) and the CM of A549 cells with a number of 2.5 × 10^6^ (lane 2), 5 × 10^6^ (lane 3), and 1 × 10^7^ (lane 4). (**C**) Comparison the levels of five miRNAs in the exosome-free supernatant and exosome-enriched pellet of the CM from A549 cells. The total expression level of each miRNA was set to 1. (**D**) Relative miR-1246 levels in the exosomes, exosome-free supernatant and the CM from A549 and H446 cells. The miR-1246 level in exosomes was set as control. Data were presented as means ± SEMs of five independent experiments (*P < 0.05,** *P* < 0.01). (**E**) Localization of exosomes and extracellular miR-1246 in the recipient A549 cells. The CM was collected from A549 cells labeled with DiO and Cy3-labeled miR-1246 and was then used to incubate other recipient A549 cells for 24 h. The recipient A549 cells with red fluorescence Cy3-labeled miR-1246, green fluorescence exosomes, and DAPI stained nuclear with blue fluorescence were fixed and observed under a confocal microscopy.

Previous studies have shown that extracellular miRNAs can be carried by exosomes and transport over long distances to its recipient cells and then induce *de novo* transcriptional and translational changes [13, 29, 30]. In order to determine whether exosome is a carrier of the extracellular miR-1246 found here, we investigated the characteristics of exosomes obtained from the CM of A549 cells. Results showed that these exosomes had a morphological uniform vesicular structure (Figure 2A) and could obviously express the exosomal marker proteins CD63 and Hsp70 (Figure 2B). Using the total exosome isolation reagents, the culture medium was separated into two fractions, an exosome-free supernatant and an exosome-enriched sediment. Although both fractions contained miRNAs, most of miRNAs had higher concentrations in the supernatant. The ratio of miRNA in the exosomes to that in the exosome-free supernatant was shown in Figure 2C. It could be seen that miR-17-5p, -24-3p and -1246 were mainly presented in the exosome-free supernatant rather than in the exosomes except that miR-2861 and miR-92a-3p had relative higher levels in the exosomes. Especially, the level of miR-1246 in the exosome-free supernatant was 5- and 2.5- times of that in the exosomes for both A549 and H446 cells (Figure 2D).

To further determine the existing form of extracellular miR-1246, we analyzed whether miR-1246 was co-localized with exosomes. A549 cells were transfected with Cy3-labeled miR-1246 with red fluorescence and then labeled with green-fluorescing lipophilic dye DiO for exosomes. After well washing to remove any extra miRNA and dyes outside cells, the cells were cultured in fresh medium for 24 h and then its CM was collected and transferred to other recipient A549 cells and incubated the cells for 12 h. Figure 2E displayed that both DiO-labeled exosomes and Cy3-labeled miR-1246 existed in the recipient cells, indicating that both exosomes and miR-1246 were released from the miRNA-transfected cells and further absorbed by the recipient cells. Moreover, the cell images clearly showed that a large amount of miR-1246 was not co-localized with exosomes but presented in a non-exosome-associated form.

### Extracellular miR-1246 can actively enter into recipient cells

**Figure 3:**
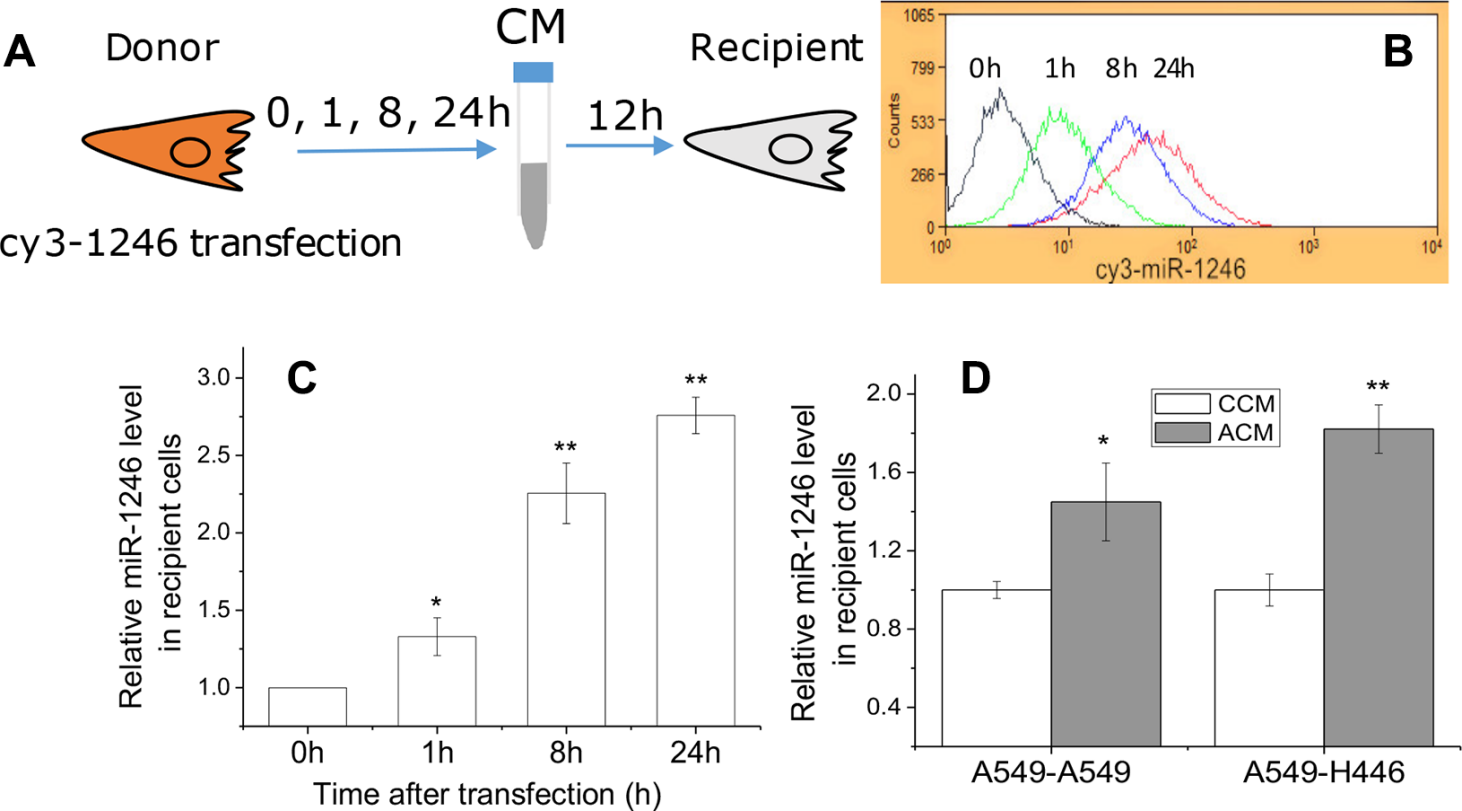
Extracellular miR-1246 was integrated into recipient cells (**A**) The scheme of experiments. A549 cells were transfected with Cy3-labeled miR-1246, then the CM was collected at 0, 1, 8 and 24 h after transfection and used to culture other recipient A549 cells for 12 h. The uptake of miR-1246 by A549 cells was detected by flow cytometry (**B**) and qRT–PCR (**C**), respectively. (**D**) Relative levels of intracellular miR-1246 in A549 and H446 cells treated with CM from non-irradiated A549 cells (CCM) or from 4 Gy X-ray irradiated A549 cells (ACM) for 12 h. The expression level of miR-1246 in the cells treated with CCM was set as control. Data were presented as means ± SEMs of five independent experiments (*P < 0.05, **P < 0.01).

Can the non-exosome associated miR-1246 be integrated into the recipient cells? To confirm this, A549 cells were transfected with Cy3-labeled miR-1246 and further cultured for 0, 1, 8 and 24 h, and the CM was collected and transferred to recipient A549 cells and maintained for 12 h (Figure 3A). The uptake situation of the miR-1246 was detected by flow cytometry and qRT–PCR, respectively. It was found that the content of miR-1246 in the recipient A549 cells and its mRNA level increased with the cell incubation time (Figure 3B and 3C), which indicates that the transfected miR-1246 has been effectively integrated in the recipient cells.

**Figure 4:**
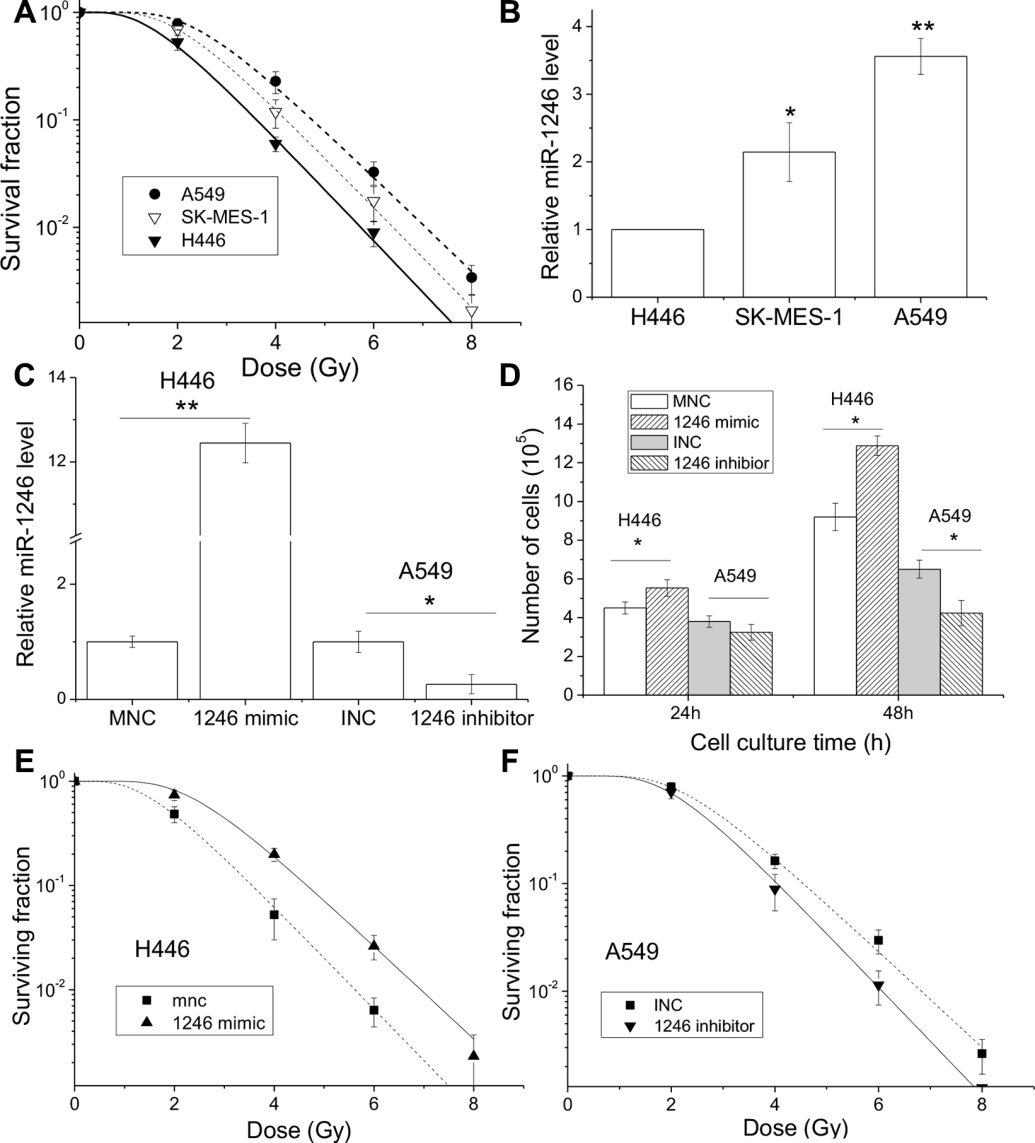
miR-1246 mimic promotes cell proliferation and enhances radioresistance Lung cancer cell lines of A549, SK-MES-1 and H446 were irradiated with X-rays. (**A**) Dose response of cell survival. (**B**) Intracellular miR-1246 expressions of lung cancer cells. (**C**) The miR-1246 expressions in H446 and A549 cells transfected with miR-1246 mimic and its negative control (MNC), miR-1246 antisense inhibitor and its negative control (INC), respectively. (**D**) Cell number of above H446 and A549 cells at 24 and 48 h after transfection. (**E**) Clonogenic survivals of H446 cells transfected with miR-1246 mimic and its control. (**F**) Clonogenic survivals of A549 cells transfected with miR-1246 inhibitor and its control. Data were presented as means ± SEMs of four independent experiments (*P < 0.05, **P < 0.01).

Moreover, we examined whether miR-1246 derived from irradiated cells could actively enter into the recipient cells. When A549 or H446 cells were incubated for 12 h with the ACM from A549 cells irradiated with 4 Gy X-rays, the levels of miR-1246 in the ACM-treated A549 or H446 cells were increased by 1.4- and 1.8- times of control, respectively (Figure 4D), which verifies that the non-exosome associated miR-1246 has entered into the recipient cells effectively.

### MiR-1246 promotes cell proliferation and radioresistance

As shown in Figure 1F, the intracellular miR-1246 has a rapid response to IR and thus may be involved in cellular radiosensitivity. It is well known that, among different subtypes of lung tumors, the adenocarcinoma and squamous carcinoma have a lower radiosensitivity compared with small-cell lung carcinoma. To evaluate the impact of miR-1246 on radiosensitivity, we evaluated the radiosensitivities and the expressions of miR-1246 in three lung cancer cell lines of A549, SK-MES-1 and H446 from lung adenocarcinoma, squamous carcinoma and small-cell lung carcinoma, respectively. Figure 4A illustrates the dose responses of the clonogenic survival of A549, SK-MES-1 and H446 cells. The D_0_ values, i.e. the mean lethal dose used to characterize the radiosensitivity, were calculated to be 0.987 Gy, 0.933 Gy and 0.907 Gy for these cell lines, respectively. Further assay showed that the intrinsic miR-1246 had the lowest level in the radiosensitive H446 cells but had the highest level in the radioresistant A549 cells (Figure 4B), suggesting that the level of intrinsic miR-1246 may positively associate with cell radioresistance.

To test the above assumption, we performed the analyses of gain-of-function and loss-of-function of miR-1246 by transferring a miR-1246 mimic into H446 cells to increase its miR-1246 level and transferring a miR-1246 antisense inhibitor into A549 cells to reduce its miR-1246 expression. Both interventions had good efficiencies in regulating miR-1246 expression in the corresponding cells (Figure 4C). At 48 h after transfection, the miR-1246 mimic strongly promoted H446 cell proliferation by about 1.4-fold of control but miR-1246 inhibitor reduced the proliferation of A549 cells by 35% (Figure 4D). Moreover, the miR-1246 mimic significantly decreased the radiation sensitivity of H446 cells (Figure 4E). The sensitization enhancement ratio (SER), the ratio of D0 of the mimic negative control (MNC) vs. miR-1246 mimic treated cells, was 0.897. Conversely, transfection of miR-1246 antisense inhibitor enhanced the radiation sensitivity of A549 cells with a SER of 1.125 (Figure 4F). In addition, we also measured the influence of another miRNA candidate, miR-2861, on the radiation sensitivity of A549 cells, but did not find any change (data not shown). Accordingly, miR-1246 does contribute to cell proliferation and leads to cell radioresistance.

### DR5 is the direct target of miR-1246 in charge of radioresistance

**Figure 5:**
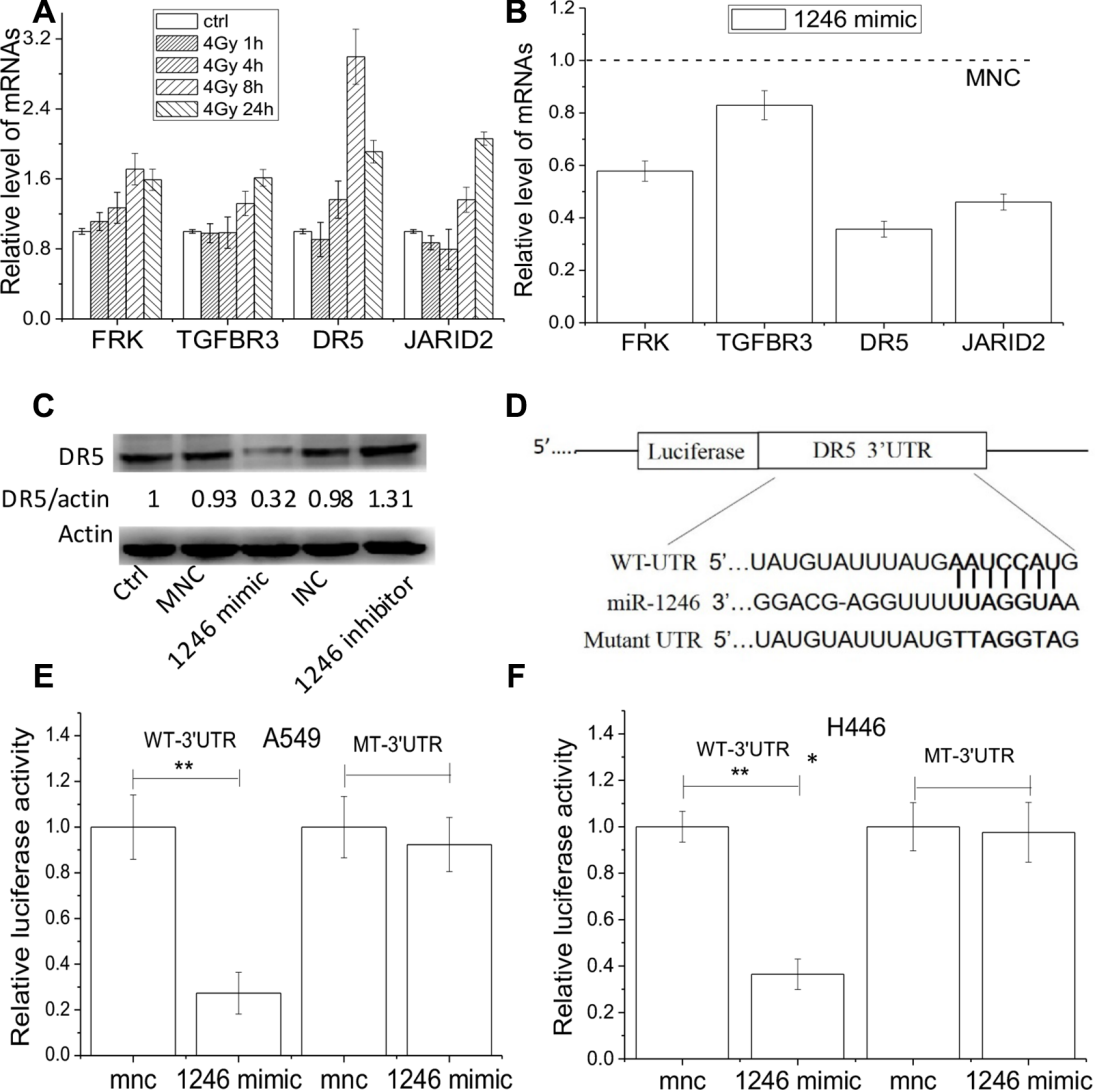
DR5 is the direct target of miR-1246 Expressions of mRNA and protein were detected by RT-pCR and Western blot assay, respectively. (**A**) Relative expression levels of JARID2, FRK, DR5 and TGFBR3 in H446 cells after 1, 4, 8, and 24 h of 4 Gy irradiation. (**B**) Relative levels of the above four genes in H446 cells transfected with miR246 mimic in comparison of MNC. (**C**) DR5 protein expressions in H446 cells treated with miR-1246 mimic, inhibitor, or negative controls. Actin was used as an internal reference. (**D**) The predicted miR-1246 binding sites in the wild-type (WT) DR5 UTR and mutant DR5 UTR. (**E, F**) WT or mutant 3′UTR- DR5 Gaussian Luciferase reporter vector was co-transfected with miR-1246 mimic or its control into A549 cells and H446 cells, and Gaussian luciferase activity of each sample was normalized to the secreted alkaline phosphatase (SEAP). Data were presented as means ± SEMs of four independent experiments (*P < 0.05, **P < 0.01).

It is generally accepted that miRNAs exert their function by down-regulating its target genes. MiR-1246 executes its survival-promoting function in cancer cells, like an oncogene, probably by inhibiting its target genes that usually have tumor suppression effect. Based on this rationale, four potential genes (JARID2, FRK, DR5 and TGFBR3) were selected from the databases of miRwalk, miRanda and TargetScan according to their potential functions in tumor suppressive processes [31–34]. The qRT-PCR assay illustrates that these four genes in H446 cells could respond to irradiation and their expressions began to increase at 8 h after IR, where DR5 was over-expressed to a much higher level (Figure 5A). However, these mRNA expressions, especially DR5, were significantly decreased in the miR-1246 mimic transfected H446 cells (Figure 5B). In concordance with these results, DR5 protein level was also down-regulated in the miR-1246 mimic treated cells, while it was upregulated in the miR-1246 inhibited cells (Figure 5C).

To disclose whether DR5 is the direct target of miR-1246, the 3′UTR of DR5 gene was inserted into a luciferase reporter vector (Figure 5D). A549 and H446 cells were co-transfected with this luciferase reporter plasmid bearing 3′UTR of DR5 and miR-1246 mimic or its negative control. It was found that miR-1246 mimic significantly inhibited the luciferase activity in both A549 and H446 cells (Figure 5E). We then mutated the binding site of miR-1246 on the 3′UTR of DR5 (Figure 5D) and found that the mutant 3′UTR of DR5 was completely refractory to miR-1246–mediated luciferase reporter repression (Figure 5E).

**Figure 6:**
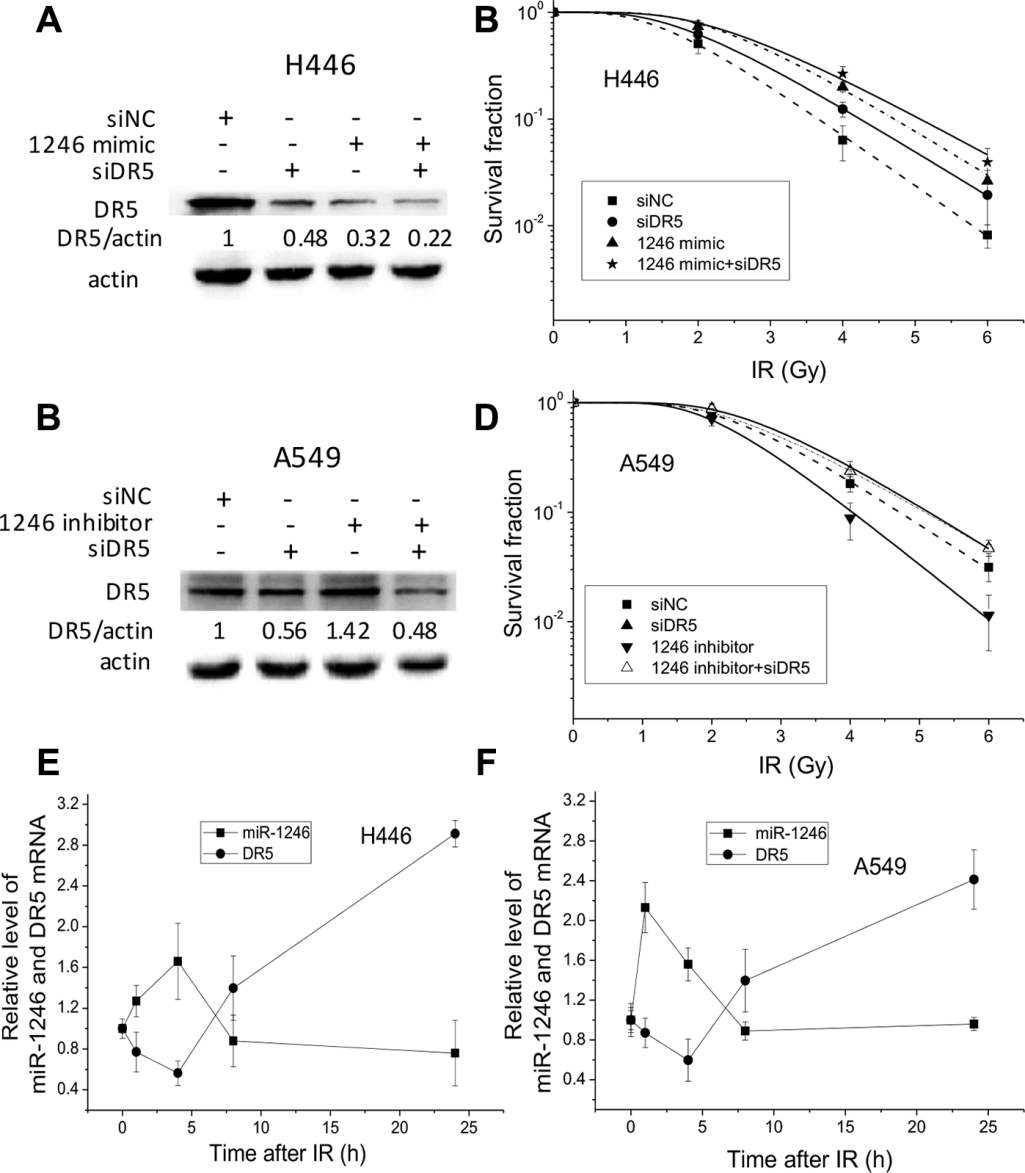
miR-1246 increases cancer cell radioresistance via directly targeting DR5 (**A**) DR5 protein expressions in the H446 cells transfected with miR-1246 mimic, DR5 siRNA and their negative controls, which were normalized to siRNA control with actin as an internal reference. (**B**) Dose response of the survival of H446 cells transfected with miR-1246 mimic, DR5 siRNA and their negative controls. (**C**) DR5 protein expressions in the A549 cells transfected with miR-1246 inhibitor, DR5 siRNA and their negative controls, which were normalized to siRNA control with actin as an internal reference. (**D**) Dose response of the survival of A549 cells transfected with miR-1246 inhibitor, DR5 siRNA and their negative controls. (**E, F**) The kinetics of miR-1246 and DR5 expressions in H446 and A549 cells after 4 Gy irradiation. Data were presented as means ± SEMs of four independent experiments.

To further understand the function of DR5 in cell survival, both H446 and A549 cells were transfected with DR5 siRNA. Western blotting assay showed that the efficiency of this siRNA interference was about 50% for both cell lines (Figure 6A and 6C). It was found that DR5 knockdown increased cell radioresistance in consistent with the function of miR-1246 mimic, and the combination treatment of cells with DR5 siRNA and miR-1246 mimic increased H446 cell survival more remarkably (Figure 6B). The SER values were 0.875, 0.827 and 0.778 for the H446 cells transfected with DR5 siRNA, miR-1246 mimic, and DR5 siRNA plus miR-1246 mimic, respectively. But miR-1246 mimic was much more effective than DR5 siRNA on enhancing cell radioresistance, suggesting that the regulation of miR-1246 on radioresistance may not only by suppressing DR5, other unknown targets are likely to be involved as well. On the other side, when A549 cells were treated with miR-1246 inhibitor, its survival fraction was effectively reduced with a SER of 1.236 (Figure 6D). But co-transfection experiments using DR5 siRNA and anti–miR-1246 showed that miR-1246 silence could not repress the radioresistance of DR5-knockdown A549 cells.

We then explored the interaction kinetics between intracellular miR-1246 and DR5 expressions in H446 and A549 cells after 4 Gy irradiation. In both cell lines, the expressions of intracellular miR-1246 increased immediately after IR until 1–4 h and then decreased to the control level rapidly. With complementary kinetics to miR-1246, the expressions of DR5 decreased rapidly after IR and then gradually increased after 4 h of IR (Figure 6E and 6F). These findings elucidate that there is a balance between DR5 and miR-1246 in regulating cell radiation sensitivity, and miR-1246 renders cancer cells radioresistant via directly targeting the DR5.

### MiR-1246 promotes proliferation and radioresistance of bystander cells by suppressing DR5

**Figure 7:**
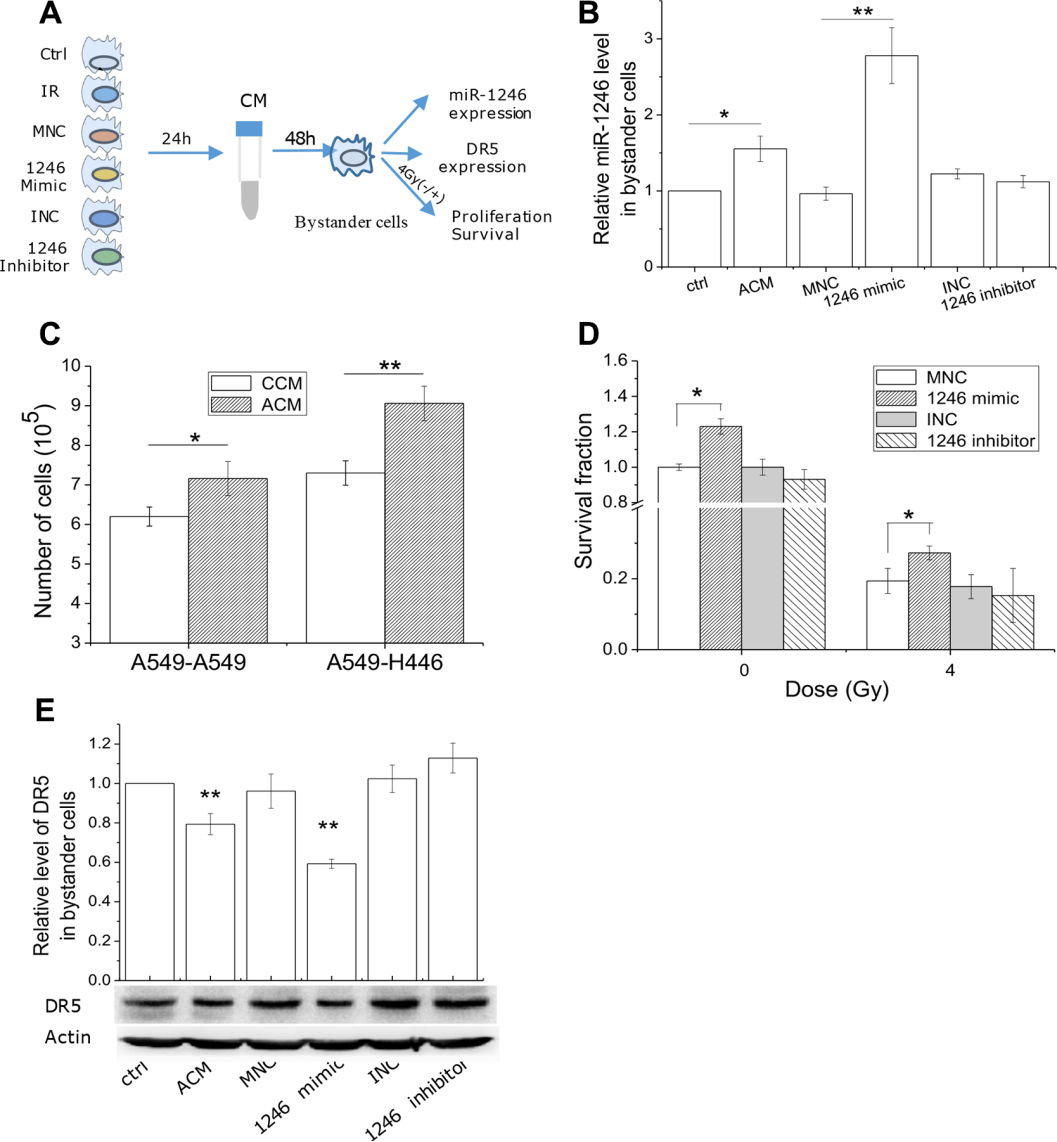
Extracellular miR-1246 promotes cell proliferation and induces radioresistance via directly suppressing DR5 in bystander cells (**A**) The scheme of experiments. A549 cells were irradiated with 4 Gy X-rays or transfected with miR-1246 mimics, miR-1246 inhibitor and their control, respectively. Then its CM was collected to treat non-irradiated bystander cells for 48 h. (**B**) miR-1246 expressions in the bystander H446 cells treated with the CM from A549 cells as shown in Figure 7A. (**C**) Proliferations of H446 and A549 cells after 48 h of incubation with the active CM (ACM) from A549 cells or its control (CCM). (**D**) Clonogenic survival of irradiated H446 cells that were treated with the CM indicated above. (**E**) DR5 protein expression in the bystander H446 cells that were treated with the CM indicated above. Data were presented as means ± SEMs of three independent experiments (*P < 0.05, **P < 0.01).

After knowing the role of intrinsic miR-1246 in cell radiosensitivity, we determined whether miR-1246 could function as a bystander signaling factor and hence influence the radiosensitivity of bystander cells once it was released from irradiated cells. Here H446 cells with a lower level of miR-1246 was applied as the bystander cells to receive the miR-1246 released from irradiated A549 cells. The schematic of this experimental design was shown in Figure 7A. The CM was collected from A549 cells under different treatments and then delivered to recipient H446 cells. It was found that, when H446 cells were treated with the ACM from irradiated A549 cells, the level of its intracellular miR-1246 was up-regulated (Figure 7B), and its proliferation was potently stimulated (Figure 7C). When H446 cells were treated with the CM from A549 cells transfected with miR-1246 mimic, the level of miR-1246 in the bystander H446 cells was further increased (Figure 7B), and the survival of bystander cells irradiated with 4 Gy X-rays become higher than that of without ACM treatment (Figure 7D). Meanwhile, the CM from miR-1246-deficient A549 cells had no influence on both intracellular miR-1246 level and the survival of recipient H446 cells after irradiation.

To clarify the mechanism of miR-1246 as a bystander signaling factor, the protein level of DR5 in the bystander cells was determined after the CM treatments. As shown in Figure 7E, the expression level of DR5 in the bystander H446 cells was potently decreased after incubation with the ACM from irradiated A549 cells and it was further reduced by the CM from miR-1246 mimic transfected A549 cells. While the treatment of cells with the CM from miR-1246 negative control A549 cells had no effect on DR5 protein expression in the bystander H446 cells. Therefore, radiation-induced extracellular miR-1246 could acts as a bystander signal molecule shuttling from irradiated cells to bystander cells and enhance the radioresistance of bystander cells by inhibiting its DR5 function.

## DISCUSSION

The profiles of miRNAs in the extracellular environments of different bio-fluids reflect the alterations of physiological and pathological progresses [4–6, 35]. A large number of studies have raised that the extracellular miRNAs may play an important role in regulating intercellular communication and thus have implications in tumor biology [13, 16, 29, 30, 36]. In addition, some evidences indicate that extracellular miRNAs are relevant to RIBE [24, 25]. However, there is no literature concerning the role of radiation-induced extracellular miRNAs in regulating cell radiosensitivity.

Our microarray measurement showed that 21extracellular miRNAs were released from irradiated A549 cells, where 6 miRNAs, let-7i-5p, miR-17-5p, -24-3p, -92a-3p, -1246 and miR-2861 were abundant and could be further up-regulated by IR. Let-7i-5p, miR-17-5p, -24-3p, -92a-3p and -1246 have been detected in the blood of cancer patients [37–41] with exosome-associated forms [27, 42–44]. If a miRNA can regulate the radiosensitivity of lung cancer cells, its concentration outside of cells should not lower than that inside of cells. As shown in Figure 1D, among let-7i-5p, miR-17-5p, -24-3p, -92a-3p, -1246, and miR-2861, only extracellular miR-1246 had a level higher than that inside of its donor cells, whereas the other five extracellular miRNAs had much lower levels. Moreover, our data showed that miR-1246 could be released from not only irradiated cells but also from non-irradiated cells in a time-dependent manner, and the changes of extracellular miR-1246 was remarkably greater than intracellular miR-1246 (Figure 1E and 1F). This confirms previous reports that most extracellular miRNAs have lower levels compared with their intracellular contents, while some miRNAs can be actively secreted from donor cells under both normal and pathological conditions and appear at relative higher levels than their donor cells [27, 28].

Many studies have demonstrated that the circulating miRNAs are packaged into exosomes [7–9] and hence able to bypass the high activity of RNases in the bio-fluids. Umezu *et al.* reported that the extracellular miR-92a in exosomes from leukemia k562 cells significantly reduced the expression of integrin-α5 in the recipient HUVEC cells and could enhance the endothelial cell migration and tube formation [15]. Other study showed that the exosomal miR-105 could destroy the tight junctions and integrity of endothelial monolayers and promoted cancer metastasis [30]. However, by using a systemic approach, this work reveals that there are at least two populations of extracellular miRNAs in the culture medium i.e. exosome-associated form and non-exosome-associated form. In fact, the extracellular miR-1246 is mainly found in the non-exosome associated from, which was confirmed by labeling the exosomes and miR-1246 with different fluorescence dyes as shown in Figure 2. These results are consistent with other reports that the majority of released miRNAs are not associated with exosomes but with protective protein complexes to prevent their degradation and thus can be functionally transferred to other recipient cells [11, 13]. Nevertheless, little is known about the mechanisms by which miRNAs are selectively secreted into the extracellular environment. Several research groups have demonstrated that the exosomes with miRNAs are released through a ceramide-dependent secretory pathway which is controlled by the enzyme of ceramide biosynthesis neutral sphingomyelinase (nSMase) and that the exosomal miRNAs can promote gene silence similar to cellular miRNAs [8, 44, 45]. Although the underlying mechanism of the transport of non-exosome associated miR-1246 remains to be fully elucidated, the shuttling phenomenon of extracellular miR-1246 between cancer cells may have potential to affect cancer cell physiological functions.

It has been reported that miR-1246 was an oncogenic miRNA linking with cancer migration and invasion [46], but there was no literature concerning the role of miR-1246 in radiosensitivity. Figure 3 shows that inhibition of miR-1246 in A549 cells bearing a high level of intrinsic miR-1246 significantly suppressed cell proliferation and increased cell radiosensitivity; while transfection of H446 cells with miR-1246 mimic promoted cell growth and enhanced cell radioresistance. Therefore, the intrinsic miR-1246 in lung cancer cells has the potential function in regulating cell radiosensitivity. This finding raises a question what is the target gene of miR-1246. Based on the analysis using some biological databases, DR5 was predicted as a target gene of miR-1246. DR5, also known as TNFRSF10b or TRAILR2, has been discovered recently and is a well-defined tumor suppressor gene that can induce cancer cell death triggered by a variety of external stresses. TRAIL and agonistic TRAIL-receptor specific antibodies are under clinical investigation for the treatment of various malignancies [47, 48].

Helwak *et al.* developed a technique for ligation and sequencing miRNA-target RNA duplexes associated with human AGO1 and validated the interactions of DR5 mRNA with miR-17-5p, -331-3p, -92a-3p and -940 [49]. Our study gives the first evidence that miR-1246 could regulate radiosensitivity via targeting DR5. Given the tumor suppressor role of DR5, it is not surprising that DR5 siRNA interference results in the up-regulation of proliferation and radioresistance in lung cancer cell lines in consistent with the effect of miR-1246. In this study, we also demonstrated that IR can regulate the expression of both miR-1246 and DR5 in lung cancer cells. The up-regulation of miR-1246 had a fast kinetics, while the pattern of DR5 expression showed a slow dynamics after irradiation. Since miR-1246 can induce radioresistance and DR5 acts as a radiosensitizer, our data suggest these is a mutual restricted relationship between miR-1246 and DR5 in balancing cell survival and death signals after exposure to IR. A common characteristic of IR is the induction of various types of DNA damage, directly leading to tumor cell death. On the other hand, DNA damage can trigger cellular protective pathways such as DNA damage repair and other pro-survival signaling. Especially, the efficient and faithful DSB repair is critical to normal cell survival but it is also a mechanism contributing to tumor cell therapeutic resistance [50]. In fact, the homeostatic balance between survival and death is a basic biological phenomenon [51]. The quick up-regulation of miR-1246 after IR could inhibit the expression of DR5 to avoid excessive cell death induced by IR. Therefore, the present study provide a new mechanism of miR-1246/DR5 regulation in the balance between cell survival and death signals when the cell is exposed to IR.

It was reported that serum miR-1246 could be up-regulated in the patients at early-stage of cervical squamous cell carcinoma [52] and the circulating miR-1246 was also increased in the patients with esophageal squamous cell carcinoma [41], multiple myeloma [53] and primary colorectal cancer [54], indicating that serum miR-1246 may be a promising biomarker for the early detection of primary cancer. Our results showed that miR-1246 was enriched outside the lung cancer cells and could be actively secreted into the culture medium in response to IR in a dose-dependent manner, and then it could be uptaken by non-irradiated bystander cells. It has been reported that large parts of extracellular miRNAs are the by-products of dead or dying cells [55–58]. A number of independent research groups have demonstrated that extracellular miRNAs entrapped within apoptotic bodies and exosomes can be transferred to recipient cells, which results in gene expression alteration and other cellular functions [44, 59–61]. Given the pro-survival function of intracellular miR-1246, one question emerges whether the radiation induced non-exosome associated miR-1246 could play a role in regulating survival and radiosensitivity of bystander cells.

The most commonly reported RIBEs have deleterious effects similar to that caused by direct irradiation [62]. However, it has been demonstrated in cultured cell lines that acquisition of radioresistance could be induced by bystander effect [63, 64]. As a bystander signaling factor, NO molecule contributed to the over-growth and radioresistance of bystander cells after co-culture with irradiated tumor cells [65, 66], indicating that the RIBE is not only detrimental to non-irradiated cells but could also be an advantageous phenomena for cells, just like the radiation-induced adaptive response (RIAR) that is the term for generally favorable biological responses or hormesis response to low dose IR [67, 68]. Our findings give further evidence of a linkage between RIBE and RIAR.

The other important finding in the present study is that the extracellular miR-1246 could down-regulate DR5 in the bystander cells and thereby enhanced cell survival and radioresistance. It has been discovered that the exosome-associated extracellular miRNAs can stimulate angiogenesis [69] and facilitate metastasis [70] in cancers and even act as immune modulators in tumorigenesis [71]. Our experimental results demonstrated that miRNAs in non-exosome associated form can also alter the gene expression in the bystander cells, which gives direct evidence that a non-exosome combined miRNA could specifically regulate radiosensitivity of the bystander cells. What is important is that the bystander stimulation effect of miR-1246 on cell proliferation and survival may result in radioresistance of the cells beyond of clinical target volume during tumor radiotherapy.

In summary, our work reveals that miR-1246 in a non-exosome-associated form can be released from irradiated lung cancer cells and act as a signaling regulator of cell-to-cell communication and integrated into the bystander recipient cells, which results in cell proliferation promotion and radioresistance by suppressing DR5 in the recipient cells. The current study extends our insights of the role of miRNAs in mediating intercellular communication during cancer radiotherapy. What's more, the newly identified miR-1246/DR5 axis sheds light on the molecular mechanism of lung cancer radioresistance, indicating that it is a potential strategies to treat radioresistant lung cancer by inhibiting miR-1246 or activating the DR5.

## MATERIALS AND METHODS

### Cell culture and preparation of conditioned medium

Human non-small cell lung cancer cell line A549, SK-MES-1 and human small cell lung cancer cell line NCI-H446 (H446) were purchased from Shanghai Cell Bank (Shanghai, China). These cells have been commonly used as a model in the mechanistic studies of lung cancer radiosensitivity [72–74], and they were cultured in RPMI-1640 medium with supplement of 10% fetal bovine serum (FBS, Gibco Invitrogen, Grand Island, NY, USA), 100 U/ml penicillin and 100 μg/ml streptomycin. All of the cells were cultured at 37°C in an atmosphere of 5% CO_2_.

To prepare the conditioned medium (CM), cells at 70% confluence were washed twice with phosphate-buffered saline (PBS) and replenished with fresh medium containing 10% exosome-free FBS (by 120,000 g ultracentrifugation overnight). The cells were irradiated using an X-ray source (X-RAD 320, Precision X-Ray, North Branford, CT, USA) operated at 300 kV and 8 mA with a 2-mm Al filter at a dose rate of 2.0 Gy/min. The CM from irradiated A549 or H446 cells, referred as active-conditioned medium (ACM), and the CM from control cultures (CCM) were collected from 0 h to 48 h post-irradiation and centrifuged at 300 × g for 3 min at 4°C to remove any floating cells, then the supernatants were centrifuged at 2,000 × g for 20 min and 12,000 × g for 60 min at 4°C and passed through a 0.22 μm filter to remove any cell debris. The CM was used for experimental analysis immediately or stored at −80°C for further analysis.

### RNA isolation and miRNA expression profile in CM

CM from irradiated and control A549 cells 24 h after IR were collected for miRNA expression profile assay. Total RNA was extracted from 8 ml CM using a mirVana miRNA Isolation Kit (Ambion Inc., TX, USA) and analyzed with a miRNA microarray kit (Release 19.0, Agilent Technologies, CA, USA) described earlier [35]. There are 2006 human miRNAs on this microarray (Design ID: 046064). Briefly, 200 ng of total RNA was labelled and hybridized using the miRNA microarray kit. Hybridization signals were detected with a DNA microarray scanner G2505C (Agilent Technologies) and the scanned images were analyzed using the Agilent feature extraction software (v10.7.3.1). Normalization was performed using the Agilent GeneSpring GX software version 12.5.

### Isolation of exosomes and non-exosomal fractions from conditioned medium

Free cell CM samples were used for exosome isolation using the Total Exosome Isolation Reagent Kit (Invitrogen, CA, USA). Briefly, 2 ml exosome isolation reagent was added to 4 ml of CM, incubated 12 h at 4°C, and centrifuged at 10000 × g for 1 h to obtain pelleted exosomes and non-exosome supernatant. The exosome pellets were suspended in 100 μl of PBS. Total RNA and proteins in the exosomes and exosome-depleted supernatant were isolated for miRNA PCR analysis and marker protein detection, respectively.

### Quantification of miRNAs by real time quantitative reverse-transcription PCR (qRT-PCR)

Reverse transcription of total RNA to cDNA was carried out in 20 μl reaction reagents of the mi *DETECT* A Track™ qRT-PCR Kit (RiboBio, Guangzhou, China). Total RNA from 4 ml CM was suspended in 20 μl of DEPC water and 5 μl RNA were retro-transcribed in cDNA. Quantitative PCR assay was carried out in a 20 μl reaction reagent containing 1μl cDNA. It was programmed for an initial denaturation step (95°C, 10min) followed by 40 amplification cycles (95°C, 10 s; 60°C, 20 s). A synthetic 21-mer RNA (25 fmol) with a sequence that does not match any known human small RNA sequence (cel-miR-39, RiboBio) was added to the CM with the lysis solution and it was used as a spike control for normalization of qPCR assay [4, 75]. The differences between cel-miR-39 control and experimental samples were calculated using the 2^−ΔΔCt^ method. To compare the profiles of extracellular miRNAs versus intracellular miRNAs from A549 and H446 cells, equal amounts of RNA (20 ng) isolated from CM and cells were analyzed by qRT-PCR. The differences between extracellular and intracellular miRNA were calculated using the 2^−ΔCt^ method. ΔCT values = Ct values of extracellular miRNA - Ct values of intracellular miRNA.

### Observation of exosome with a transmission electron microscopy

20 μl exosome suspension was placed on a sheet of parafilm. A carbon-coated copper grid was floated on the drop for 10 seconds. Then, the grid was removed and excess liquid was drained by touching the edge of the grid against a piece of clean filter paper. The grid was touched onto a drop of 2% phosphotungstic acid (pH 7.0) for approximately 5 seconds and the excess liquid was drained off. The grid was allowed to dry for several minutes and then examined using a JEM-1200 EX microscope (JEOL, Akishima, Japan) at 80 kV.

### Shuttling assays for fluorescently-labeled exosomes and miR-1246

A549 cells were transfected with 50 nM of Cy3-labeled miR1246 with red fluorescence using Lipofecamine^®^3000 reagent (Invitrogen). After 6 h of transfection, cells were washed with PBS triply and further labeled with 10 mM of DIO, a green-fluorescing lipophilic membrane dye, for 30 min and subsequently washed with PBS triply to remove any excess dye. After incubation for 0 h, 1 h, 8 h and 24 h, 2 ml of the culture medium were harvested and transferred to recipient A549 cells seeded at a density of 1 × 10^5^ per well in a 35 mm confocal dish. After 12 h, these cells were stained with DAPI. The fluorescence images of Cy3-labeled miR1246 and DIO-labeled exosomes in the recipient cells were observed with a confocal microscopy (Olympus FV1000, Japan).

### Flow cytometry assay

A549 cells were seeded in 6-well plates at a density of 2 × 10^5^ per well for overnight and then transfected with 50 nM of Cy3-labeled miR1246 as described above. At 6 h after the transfection, the triply washed cells were cultured with serum-free RPMI-1640 medium for 0 h, 1 h, 8 h and 24 h, then the media were harvested and transferred to the recipient A549 cells seeded at a density of 5 × 10^5^ per dish one day before. After 12 h incubation, the recipient A549 cells were harvested and analyzed using a flow cytometry (Gallios, Beckman, USA).

### Prediction and quantitative analysis of miR-1246 target gene

MiRwalk, miRanda and TargetScan were used to predict miR-1246 target genes. The miRNA target genes function were analyzed by the DAVID Bioinformatic Resource (http://david.abcc.ncifcrf.gov/). Based on the potential pathways involved in cell proliferation and apoptosis, four target genes (JARID2, FRK, DR5 and TGFBR3) were selected for RT-PCR analysis after transfection of miR-1246 mimic in H446 cells. The respective primers sequence were obtained from the Primer Bank (http://pga.mgh.harvard.edu/primerbank/). Total RNA was isolated using the E.Z.N.A.^®^ Total RNA Kit (Omega Bio-Tek, Inc., GA, USA). The expression levels of indicated genes were quantified using RT-PCR analysis. In brief, RNA was transcribed using a QuantiTect reverse-transcription Kit (QIAGEN, Shanghai, China), and the quantitative assessments of cDNA amplification for each gene were detected with a QuantiTect SYBR Green PCR Kit (QIAGEN) by a Stratagene MX3000P Cycler (Agilent). The dissociation curve (melting curve) for each gene was performed to verify the quality of the primes. Gene expression was normalized to the Ct value of b-actin from the same sample and the relative expression level of each mRNA was analyzed using a comparative CT (2^−ΔΔCT^) method.

### MiR-1246 mimic, inhibitor, and DR5 siRNA transfection

A549 and H446 cells were plated at a density of 2 × 10^5^ per well and allowed to adhere overnight. When the cell cultures were at about 70% confluence, the medium was replaced with antibiotic-free RPMI-1640. Then H446 cells were transfected with 50 nM of hsa-miR1246 mimics or its negative control (RiboBio) and A549 cells were treated with 100 nM of has-miR-1246 antisense inhibitor or its negative control (RiboBio) using Lipofecamine®3000 reagent. The transfection efficiency was evaluated by qRT-PCR at 48 h after transfection.

To investigate the effect of loss-of-DR5 on radiosensitivity of lung cancer cells, siRNA-mediated knockdown of DR5 was performed. A549 and H446 cells were transfected with 100 nM DR5 siRNA or its negative control (GenePharma, Shanghai, China) using Lipofecamine^®^3000 reagent. The transfection efficiency was evaluated by Western blot assay at 48 h after transfection. The survival of DR5 siRNA transfected cells were measured with a colony formation assay.

### Colony formation assay

After the transfection of hsa-miR1246 mimics/inhibitor, DR5 siRNA, or their negative control, A549 and H446 cells were re-plated for the measurement of radiation survival curves by using colony formation assay. Identified number of cells were re-seeded into 6-well plate and incubated for overnight allowing cell attachment, then the cells were irradiated with 0–8 Gy of X-rays, further incubated at 37°C for 2 weeks, fixed with methanol for 20 min, and stained with 0.1% crystal violet for 30 min in order to count the colonies.

### Plasmid construction and luciferase assay

To verify whether DR5 is a direct target of miR-1246, we constructed the 3′UTRs of DR5 gene into a Gaussia luciferase reporter vector pEZX-MT05 (Genecopoeia, USA). The plasmid was designed on the basis of the sequence of miR-1246 binding sites. 8 × 10^4^ A549 and H446 cells were seeded in a 24-well plate and grew to about 70% confluence, the cells were co-transfected with has-miR-1246 or its negative control and the constructed pEZX-MT05 luciferase report. On the other side, the predicted binding site of miR-1246 on the DR5 3′UTR was mutated to evaluate the influence of miR-1246 on the expression of reporter gene. The Gaussia Luciferase activities of cell culture medium collected 48 h after transfection were measured with a plate reader (Infinite M200Pro, Tecan Co., ZH, Switzerland) at 480 nm using the Secrete-PairTM Dual Luminescence Assay Kit (Genecopoeia, MD, USA). Using the secreted alkaline phosphatase (SEAP) signal as an internal standard control to eliminate the impact of cell number variations and EF1A-PG04 media as a positive control to test the illuminometer. Data were calculated with the normalized GLuc activity (GLuc/SEAP ratio) of all samples.

### Western blotting assay

Total cellular and exosome proteins were extracted using SDS lysis buffer (250 nM Tris-HCL, pH 7.4, 2.5% SDS) with 100 mM phenylmethanesulfonyl fluoride (PMSF) (Beyotime). Equal amounts of proteins (25 μg/sample) were separated with 10% SDS-PAGE and transferred onto a PVDF membrane, followed by probing overnight at 4°C with antibodies against DR5 (1:500; Abcam), CD63 (1:1000; Santa Cruz Biotechnology), and Hsp70 (1:1000; ProteinTeck). The membranes were then incubated for 1 h with the secondary HRP-conjugated antibodies (1:1000; Cell Signaling Technology). Monoclonal rabbit b-actin antibody (1:1000; Abcam) was used as an internal control. Proteins in the membrane were detected by the enhanced chemluminescence system (ECL kit, Millipore) and its band images were analyzed with the Bio-Rad ChemiDoc XRS system (BIO-RAD, USA).

### Statistical analysis

Data were presented as means ± SEMs of at least three independent experiments. Student's *t*-tests are used to perform statistical analysis and *P* < 0.05 was considered as significant difference between indicated groups.
